# Analysis of the Multiplexing Method of New System Navigation Signals of GPS III First Star L1 Frequency in China’s Regional

**DOI:** 10.3390/s19245360

**Published:** 2019-12-05

**Authors:** Hongjun Ye, Xiaojun Jing, Liang Liu, Shuo Hao, Baoguo Yu

**Affiliations:** 1 School of Information and Communication Engineering, Beijing University of Posts and Telecommunications, Beijing 100876, China; jxiaojun@bupt.edu.cn; 2 State Key Laboratory of Satellite Navigation System and Equipment Technology, Shijiazhuang 050081, China; 15097335662@163.com (L.L.); alizhuomian@163.com (S.H.); yubg@sina.cn (B.Y.)

**Keywords:** GPS III, new system signal, multiplexing method

## Abstract

Compared with the previous GPS satellites, the first GPS III satellite adds a new civil signal L1C to the signal components of the L1 frequency in addition to the improvement of positioning accuracy, anti-interference ability, and service life. The selection and combination of signal modulation and multiplexing methods will affect the power ratio and phase relationship in the process of signal transmission. In the distribution of constellation of different modulation modes, the signal amplitudes of different signal constellation points will be affected by the nonlinear amplifiers of satellites. The analysis can assess its impact on navigation performance. The iGMAS monitoring and evaluation center of the 54th Research Institute of China Electronics Technology Group Corporation uses the low-distortion data acquisition and processing platform and refined signal software receiving processing algorithm of the iGMAS monitoring and evaluation center to complete the signal acquisition of the first satellite of GPS III over China, and processes accordingly for its signal modulation mode. Compared with the previous generation GPS of old system signals, it is found that the GPS signal of the new system not only adds the L1C frequency, but also the constant envelope multiplexing mode of the L1 frequency signal, and the power ratio of the internal signal components are also adjusted.

## 1. Introduction

In order to improve the accuracy and reliability of GPS for users, in 1998, US Vice President Al Gore issued an announcement to upgrade the GPS. In 2000, the US Congress approved this work, which is called GPS III. The first GPS III satellite was launched on 24 December 2018. Compared with the previous GPS satellites, the first GPS III satellite adds a new civil signal L1C to the signal components of the L1 frequency in addition to the improvement of positioning accuracy, anti-interference ability, and service life. The new civil signal L1C is used as the fourth civil signal after L1C/A, L2C, and L5 [1,2,3]. The new L1C signal is compatible with Beidou and GALILEO navigation signals.

With the increasing demand for satellite navigation in civil and military applications, navigation systems are required to add new navigation signals while being compatible with existing navigation signals. Since navigation frequency resources are limited, more signals need to be modulated at the same frequency. However, the navigation load is a power-constrained system. Considering the nonlinear characteristics of the power amplifier, the selection and combination of signal modulation and multiplexing methods will affect the power ratio and phase relationship in the process of signal transmission [4]. Moreover, in the distribution of the constellation of different modulation modes, the signal amplitude corresponding to different signal constellation points will be affected by the nonlinearity of the satellite power amplifier [5]. Therefore, the general practice is to implement constant envelope modulation of multiple signals at the baseband modulation end to reduce the nonlinear distortion of the signal after the power amplifier. For example, the early GPS broadcasts two components at one frequency point: military signals and civilian signals. Quadrature Phase Shift Keying (QPSK) modulation technology can achieve constant envelope transmission. Due to the needs of modernization, GPS III needs to broadcast the M code signal and the new L1C signal in addition to the original L1C/A code and P(Y) code signal at the L1 frequency point.

In the GPS IIF, the Coherent Adaptive Sub-Carrier Modulation (CASM) is used for multiplexing modulation, which ensures the constant envelope multiplexing of the L1C/A, P(Y), and L1M signals. However, the new system’s GPS III signal adds a new signal L1C, which includes two signal components, L1Cp and L1Cd. Therefore, the generation principle and the signal component power composition of the new signal multiplexing modulation method are particularly concerned. Considering that the transmission power of the navigation signal is low, it is not necessary to use a general GPS receiving device to research the modulation multiplexing method of the new signal. In order to complete the research on new signal modulation and multiplexing methods, only large-caliber antennas can be used to track the narrow beams of satellites in real time to strip off the effects of other satellite signals and noise and perform a low distortion acquisition and fine-tuning analysis with corresponding data processing software.

The iGMAS Monitoring and Evaluation Center (Shijiazhuang), the State Key Laboratory of Satellite Navigation Equipment and System Technology of CETC54, completed the signal acquisition of the first satellite of the GPS III with the 15 m large-diameter parabolic antenna of the central signal quality monitoring system. The L1 frequency signal of the old GPS system and the signal of the first satellite of the GPS III were compared and analyzed. The signal modulation mode, signal component composition, and multiplexing mode were analyzed too.

## 2. New Signal Analysis of GPS III Satellite L1 Frequency

**Figure 1:**
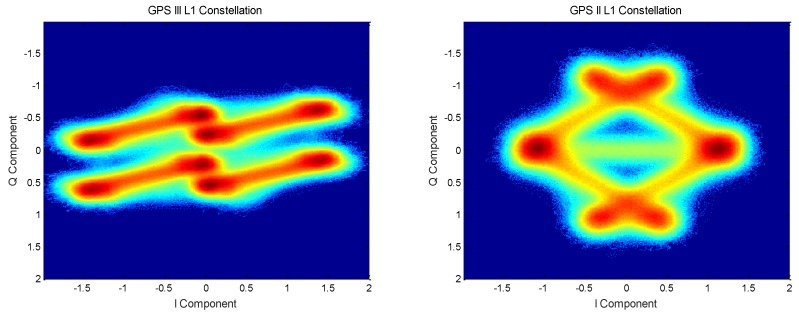
Comparison of two generations of GPS satellite L1 signal constellations.

By comparing the L1 frequency signal constellation of GPS III satellites and GPS II satellites shown in Figure 1, it can be clearly seen from the constellation diagram that the new system signal does not exhibit constant envelope multiplexing because constant envelope multiplexing is not used and the main constellation points are not distributed on the unit circle [6,7,8]. Modern Beidou and Galileo satellites use the constant envelope modulation method [9,10], which makes the signal envelope of the satellite launch constant envelope signals, thus reducing the distortion caused by the nonlinearity of the satellite transmitting power amplifier [11].

The signal elimination method is used to study the component composition and multiplexing mode of GPS III satellite signals. The basic principle of the signal elimination method is to measure the power ratio of each signal component one-by-one from the normalized energy signal. The limited transmission bandwidth of the signal (30.69 MHz) is filtered. The power ratio is the percentage of the total power of each signal component after filtering. Completing the reconstruction of each signal component and subtracting the corresponding reconstructed signal. Narrow beam directional antennas only capture one single satellite signal. This reduces the effects of ambient noise and interference and ultimately enables a fine analysis of signal modulation and multiplexing.

The acquisition of the civil reference signal is performed on the acquired signal data of the GPS III satellite L1 frequency point. The reading start point of the data is determined. The original signal of the integration period length is sequentially read according to the starting point. The reading is completed by using the DLL and the PLL tracking loop. The tracking processing of the signal obtains an accurate carrier Doppler and code phase. The observation result of the tracking loop is used to obtain the zero intermediate frequency signal with the Doppler, and the residual code phase stripped off. The bandwidth filter is transmitted according to the observed frequency. The energy normalization process is performed to obtain a final refined signal quality test signal.

The signal quality test signal is correlated with the local pseudo code to obtain a correlation peak corresponding to the signal frequency. In order to accurately obtain the energy of each component of the GPS signal within the signal transmission bandwidth, the correlation signal is filtered by a 30.69 MHz bandwidth and energy normalized, so that the square of the correlation peak-to-peak represents the power proportion of the signal component in the transmission bandwidth.

### 2.1. Recovery and Stripping of L1C/A Signal 

**Figure 2:**
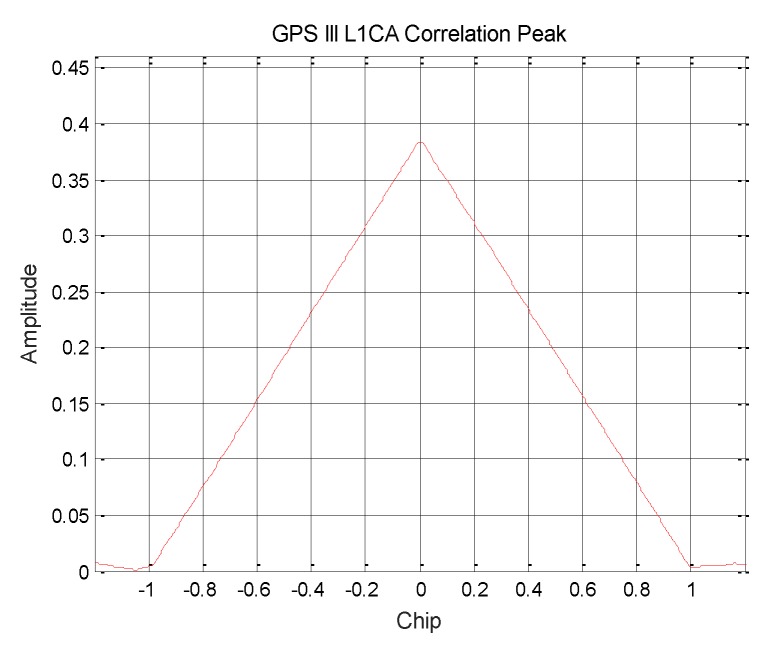
Correlation peak of GPS III satellite L1C/A signal.

On the L1C/A signal component, it can be seen from the Figure 2 that the L1C/A modulation mode is still BPSK(1), and the signal power ratio of GPS III L1C/A is about 14.75%.

**Figure 3:**
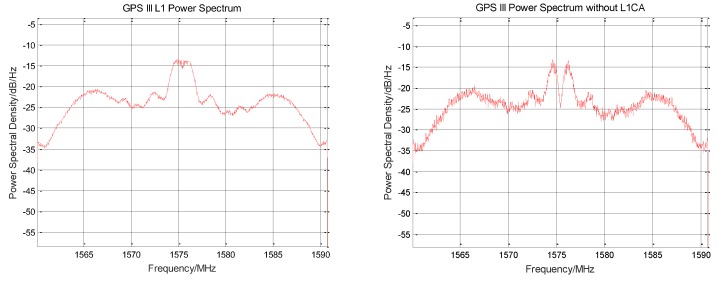
Comparison of the power spectra before and after GPS III signal stripping L1C/A signal.

The L1C/A original signal is removed from the normalized zero intermediate frequency signal, and the signal power spectrum before and after rejection is shown in Figure 3. The power spectrum shape corresponding to the BOC (1,1) modulation method used by the L1C signal component can be clearly seen from the figure, and the weak envelope of the BOC (6,1) component is seen at 1575.42 MHz ± 6 MHz. The recovered L1Cp signal cannot be completely consistent with the original signal. After subtracting the reconstructed signal, a certain noise is generated. The increase in noise can be seen on the power spectrum. 

### 2.2. Recovery and Stripping of L1C/A Signal

**Figure 4:**
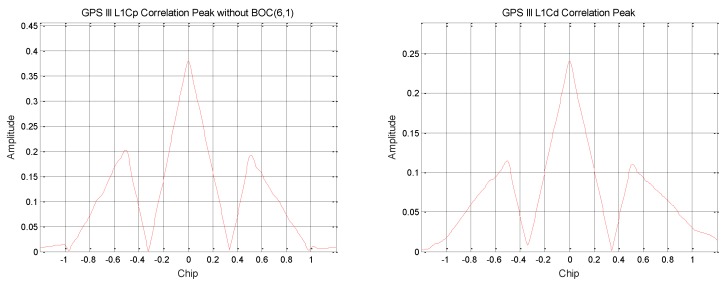
GPS III satellite L1C signal correlation peak.

By using the pseudo code of L1C to correlate according to the modulation mode of BOC (1,1), it can be found that the GPS III signal broadcasts the L1Cp (L1C pilot) signal and the L1Cd (L1C data) signal [12,13], which is shown in Figure 4. In the signal amplitude, the L1Cp signal power ratio is about 14.49%, and the L1Cd signal power ratio is about 5.80%.

**Figure 5:**
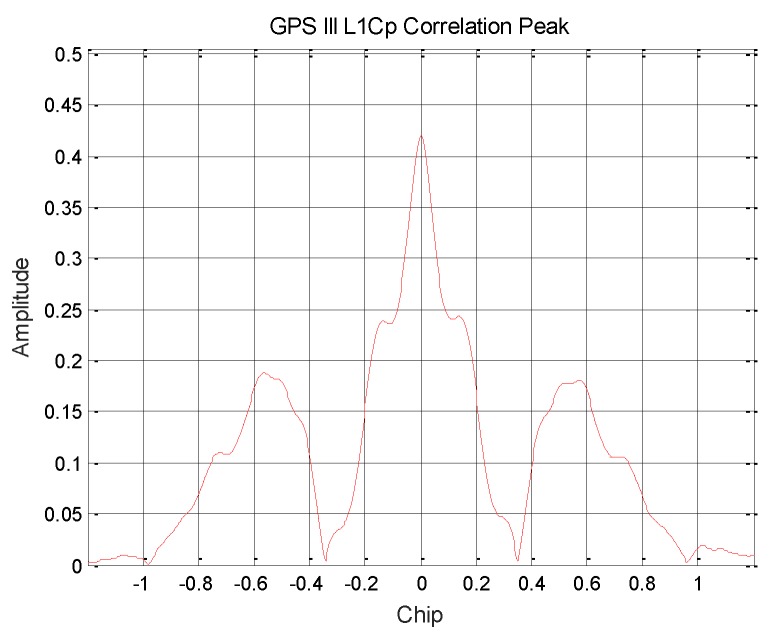
GPS III satellite L1Cp signal correlation peak.

The L1Cp signal pseudo code [14] is generated by the TMBOC modulation method. A correlation curve with a higher correlation peak-to-peak value is obtained, as shown in Figure 5. Therefore, it can be determined that GPS III broadcasts the modulation signal of TMBOC, and the power ratio is about 17.71%. According to the previously calculated result, the power ratio of the BOC (1,1) component is 14.49%, and the power ratio of the BOC (6,1) component is about 3.22%.

According to the distribution of the real part imaginary part of the L1C signal correlation peak, it can be measured that the L1Cp and L1Cd phases are on the I branch, that is, 90° out of phase with the L1C/A phase. The L1C signal can be refactored by using the power ration of The L1Cp signal (including the BOC (1,1) and BOC (6,1) components) and the L1Cd signal. The L1Cp and L1Cd can be removed from the remaining zero intermediate frequency signals in the previous step. 

**Figure 6:**
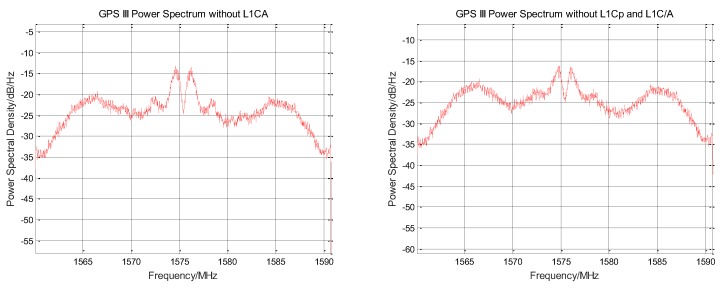
Comparison of the power spectra before and after GPS III signal stripping L1Cp signal.

The signal power spectrum before and after the elimination of L1Cp is shown in Figure 6. It can be seen from the figure that the power spectrum shape of the BOC (1,1) component is severely weakened. The envelope of the BOC (6,1) component at 1575.42 ± 6 MHz is no longer obvious.

**Figure 7:**
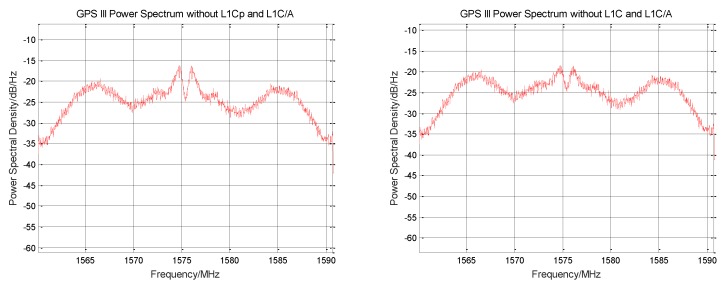
Comparison of the power spectra before and after GPS III signal stripping L1Cd signal.

The signal power spectrum before and after the elimination of L1Cd is shown in Figure 7. It can be seen from the figure that the power spectrum shape of the BOC (1,1) component is further reduced. Two sharp signal envelopes are seen near 1575.42 MHz. Since the L1C signal has been completely removed, this envelope belongs to the intermodulation component.

### 2.3. Recovery and Stripping of L1M Signal

**Figure 8:**
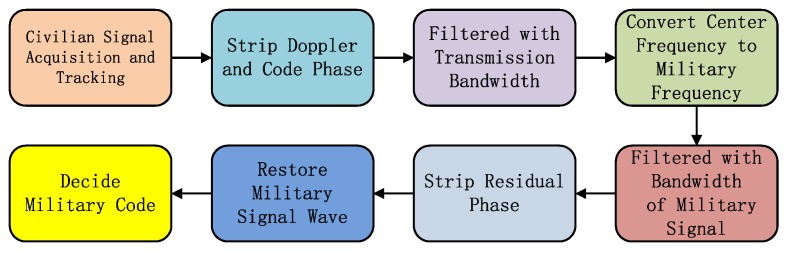
Navigation authorization code length code stream, blind identification technology.

On the L1M signal component, the gain of the acquired signal scene is high, so the blind identification method can be used to complete the decision of the M code stream and to draw the corresponding correlation peak. The process of blindly identifying long codes is shown in Figure 8. The L1M signal itself is modulated by a high-order BOC. The signal is distributed in the frequency band of 1565.19 ± 5.115 MHz and 1558.65 ± 5.115 MHz [15]. The signal is converted to a frequency of 1858.65 MHz (upper sideband). Then, the signal can be filtered by the main lobe of the signal of the upper sideband, which is 10.23 MHz. At this moment, the signal can be regarded as a BPSK modulation signal. After removing the carrier phase of the signal residual, the waveform recovery of the long code pulse signal can be completed. Finally, the optimal decision position is determined according to the code rate of the pseudo code, and the blind identification of the long code stream is completed.

**Figure 9:**
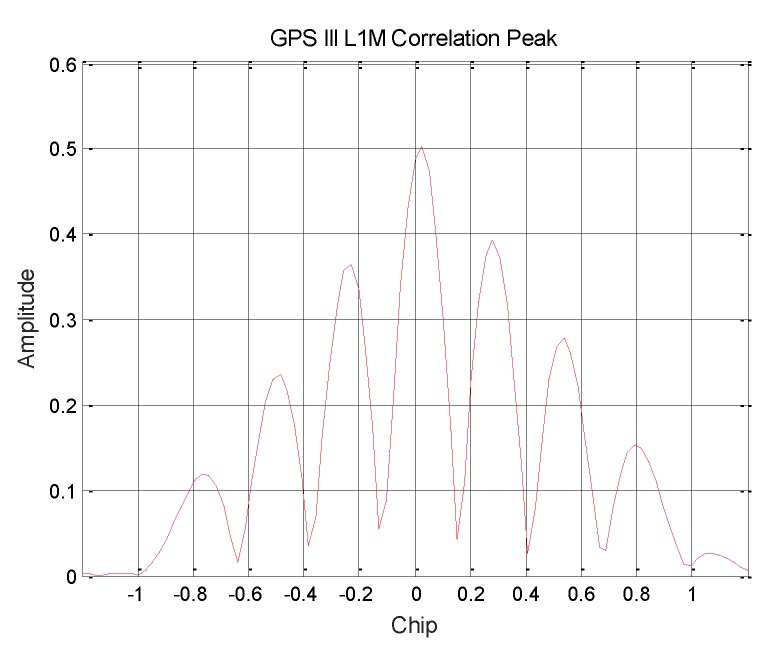
GPS III satellite L1M signal correlation peak.

It can be seen from the Figure 9 that the signal modulation mode is still BOC (10,5), and the signal power ratio of GPS III L1M is about 25.32%.

**Figure 10:**
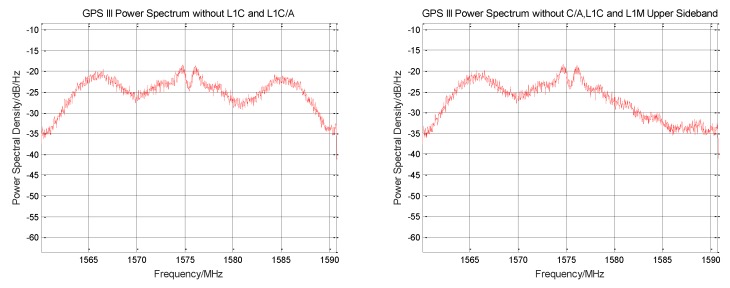
Comparison of the power spectra before and after the sideband of the GPS III signal stripping L1M signal.

The L1M modulation method, BOC (10,5), is complex, and in order to eliminate the influence of the L1M signal as much as possible, the elimination of the sideband signal on the L1M should be completed first. The comparison of the power spectrum before and after the sideband of the GPS III signal stripping L1M signal is shown in Figure 10. It can be seen from the figure that the sideband signal on the L1M has been substantially removed.

**Figure 11:**
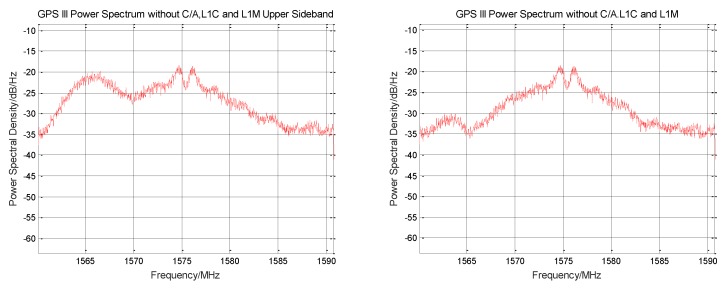
Comparison of the power spectra before and after the sideband of the GPS III signal stripping L1M signal.

The L1M lower sideband signal is stripped of the residual signal. The comparison of the power spectrum before and after the sideband of the GPS III signal stripping L1M signal is shown in Figure 11. As can be seen from the figure, the lower sideband signal has been almost removed.

### 2.4. Recovery of L1P (Y) Signal

**Figure 12:**
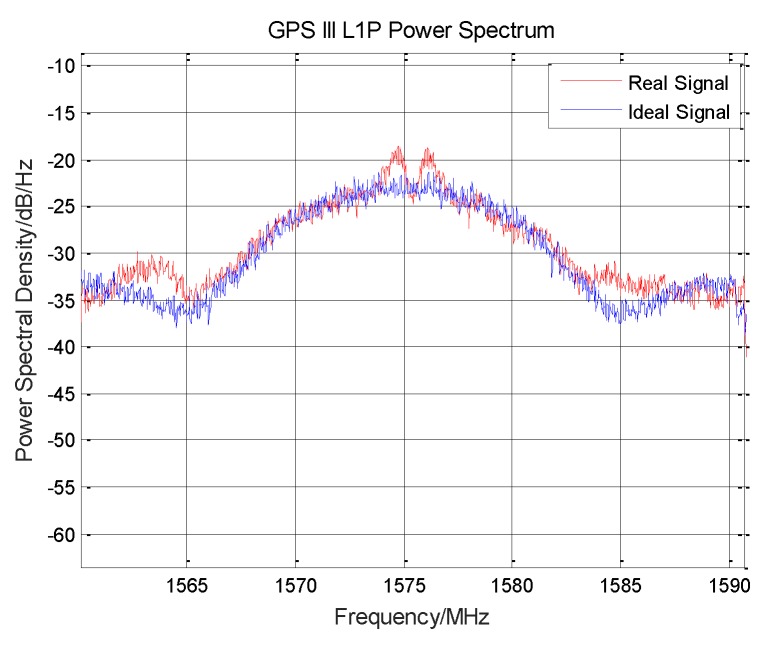
GPS III L1P (Y) signal power spectra comparison chart.

After the culling of the known signal components, the influence of each signal on L1P(Y) can be reduced. The pseudo code of L1P(Y) can be extracted from the final signal, which is shown in Figure 12. So, the correlation peak of the L1P(Y) signal and the corresponding proportion of power can be obtained.

From the power spectrum envelope of the residual signal, it is possible to vaguely see the existence of a pseudo-code signal power spectrum with a bandwidth of 20.46 MHz and a code rate of 10.23 MHz.

**Figure 13:**
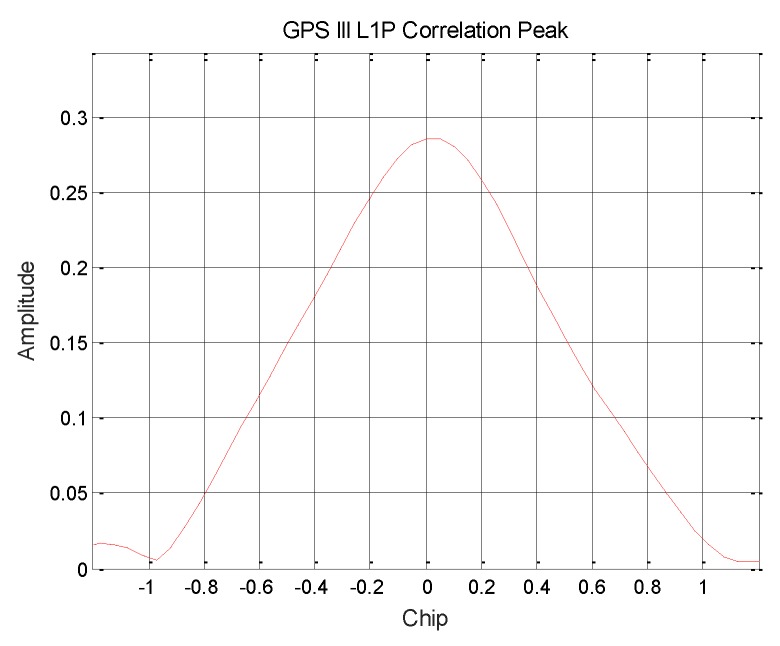
GPS III satellite L1P (Y) signal correlation peak.

After stripping the other signal components, the signal can be processed. The decision extraction of the L1P(Y) pseudo code stream in the residual signal can be completed by using the above-mentioned blind identification method. P(Y) in the observed data segment can be obtained. The code stream is correlated with the original power normalized signal to obtain the correlation peak of the L1P(Y) signal, which is shown in Figure 13. And the measured L1P (Y) signal is in the I branch, which is in phase with the L1C signal, and the power ratio of the L1P (Y) signal is about 8.17%.

## 3. Analysis of GPS III L1 Frequency Signal Multiplexing

According to the analysis results in the previous section, the composition and power ratio of each component of the GPS new system signal can be obtained. In the L1 frequency power ratio, compared with the old system GPS signal, the signal power composition of the two signals is very different, which is shown in Table 1. After the new signal component L1C is added, the corresponding power ratio of L1C/A and L1P(Y) becomes smaller. The reuse efficiency of the old and new systems is not much different. The main reason is that the new system signal needs to include L1P(Y), L1Cp, and L1Cd signals on the I branch. It is necessary to add corresponding intermodulation components to ensure the three signals and the L1C/A signals of the Q branch to synthesize the modulation method of UQPSK.

**Figure 14:**
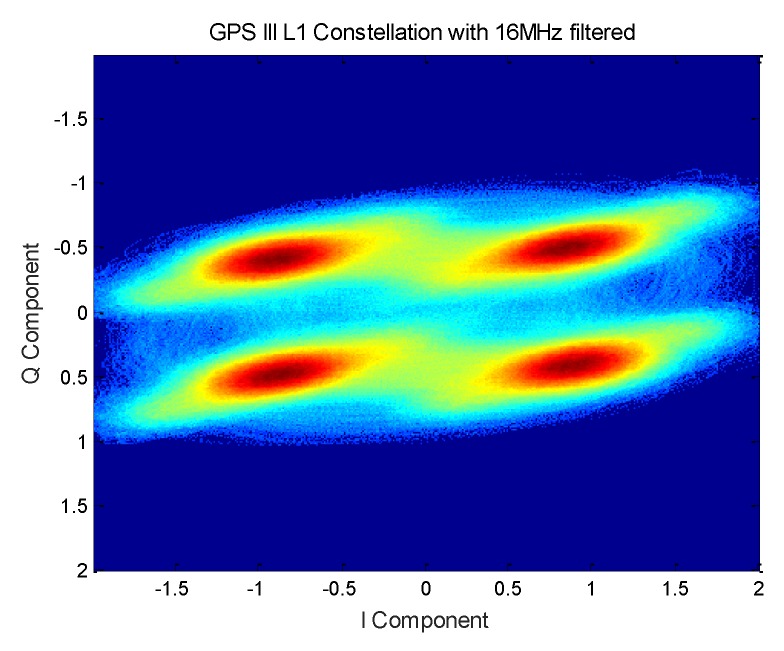
GPS III satellite 16 MHz filtered constellation.

Further analysis of the composition of the constellation of each signal component reveals that when the signal is filtered by the 16.368 MHz bandwidth at the 1575.42 MHz frequency, the presented signal constellation is shown in Figure 14.

**Figure 15:**
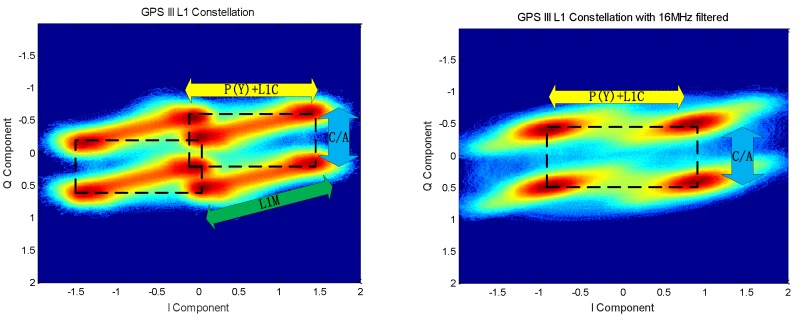
Analysis of GPS III satellite multiplexing.

Therefore, when the L1M signal is filtered out, the GPS signal is a standard Unbalanced Quadrature Phase Shift Keying (UQPSK) modulated signal. Further analysis of the signal obtained by the reconstruction and the distribution of the constellation reveals three conclusions, which are shown in Figure 15. First, the constellation strip is composed of the L1M chip and the message. Second, the up and down distribution of the Q branch is determined by the chip of the C/A code and the positive and negative of the message. Third, the left and right distribution of the I branch is determined by the chip of the P(Y) and L1C chips and the positive and negative of the message.

On the I branch, the signal distribution is binarized. However, it is determined by the three signals (L1Cd, L1Cp, and P(Y)), and the power of three signals are inconsistent because of the non-uniform weighted majority voting complex. By means of approximate time division multiplexing of three signals, the basic construction principle is to construct the multiplexed code signal C_Maj_ (t) first.

$$C_{\mathrm{Maj}}\left(t\right)=C_{L1\mathrm{Cp}}\left(t\right)\cdot C_{L1\mathrm{Cd}}\left(t\right)\cdot C_{P}\left(t\right)$$

**Figure 16:**
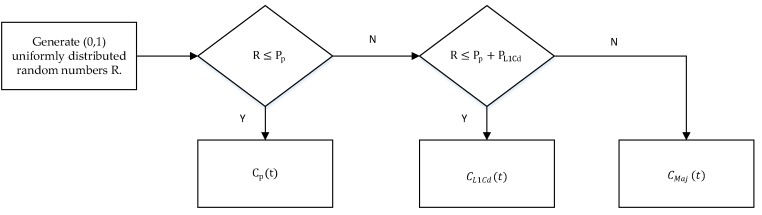
Non-uniform weighted multiplexing method basic flow.

The basic flow of the non-uniform weighted multiplexing method is as follows. Select the pseudo-code and the multiplexing code of the two weakest signal power ratios as the object of time-division output. The power ratio of L1Cd, L1Cp, and P(Y) are 5.80%, 17.71%, and 8.17%, so C_L1Cd_ (t), C_P_ (t), and C_Maj_ (t) are selected for time-division multiplexing. The output criterion of time-division multiplexing is to allocate the random number to the total power percentage interval of each signal, thereby completing the selection of the current chip output component. The basic principle is shown in Figure 16.

$$\mathrm{With}\;P_{p}=\frac{8.17}{8.17+17.71+5.8}=0.2579,P_{L1\mathrm{Cd}}=\frac{5.8}{8.17+17.71+5.8}=0.1831.$$

The non-uniform weighted majority voting multiplexing method performs LPS, L1Cd, and L1P(Y) three-way signals and L1C/A signals for QPSK multiplexing modulation, and acts as a single transmission, which satisfies the conditions of constant envelope modulation, reduces the distortion of the signal, and avoids the degradation of the effective signal power due to too many signal multiplexing paths and the effects between signals or between signals and intermodulation components.

Based on the above analysis results, the GPS III satellite signal is divided into three main signal components, namely, C/A code signal, P(Y) + L1C signal, and L1M signal. It can be clearly seen from the figure that the C/A and L1C + P(Y) signals form the UQPSK modulation, and the power ratio is about 2:1 according to the power ratio observed in the previous section. The positive and negative of the L1M signal completes the oblique shift of the UQPSK constellation. For users who only pay attention to the civilian frequency, the GPS III transmit signal is a constant envelope modulation.

The phase difference between the C/A and L1C + P(Y) signals is 90 degrees, and the UQPSK modulation is constituted. Since the square of the amplitude is proportional to the power, the combined signal of C/A and L1C + P(Y) can be written as:

$$S_{CP}\left(t\right)=\sqrt{P_{ca}}D_{ca}\left(t\right)C_{ca}\left(t\right)+j\sqrt{P_{p}}Multi\left(D_{p}\left(t\right)C_{p}\left(t\right),C_{L1CP}\left(t\right),D_{L1Cd}\left(t\right)C_{L1Cd}\left(t\right)\right).$$

P_ca and P_p are the power of the signals of the two branches of I and Q. D_ca (*t*), D_p (*t*), and D_L1Cd (*t*) are the messages of C/A code, P(Y) code, and L1Cd. C_ca (*t*), C_p (*t*), C_L1Cp (*t*), C_L1Cd (*t*) are pseudo-codes of C/A code, P(Y), L1Cp, and L1Cd signals. Multi() is a non-uniformly weighted majority voting multiplex operator. Considering that the chip and message of the L1M signal are positively and negatively correlated and that the UQPSK constellation diagram is moved in two different directions, it is assumed that the vector to be moved is: w = A + j × B, and |w| = 1. We also assume that when the product of the chip and message of the LIM signal is 1, the S_CP (*t*) vector is added by w, and when the product of the chip and message of the LIM signal is −1, the S_CP (*t*) vector is reduced by w. So, the L1 frequency signal can be written as:

$$S_{L2}\left(t\right)=S_{CP}\left(t\right)+\sqrt{P_{M}}\cdot \left[\left(D_{M}\left(t\right)C_{M}\left(t\right)\cdot 0.5+0.5\right)\cdot w+\left(-D_{M}\left(t\right)C_{M}\left(t\right)\cdot 0.5+0.5\right)\cdot \left(-w\right)\right].$$

It can also be written as:

$$S_{L1}\left(t\right)=\sqrt{P_{c}}D_{c}\left(t\right)C_{c}\left(t\right)+j\sqrt{P_{p}}Multi\left(D_{p}\left(t\right)C_{p}\left(t\right),C_{L1CP}\left(t\right),D_{L1Cd}\left(t\right)C_{L1Cd}\left(t\right)\right)\;+\sqrt{P_{M}}D_{M}\left(t\right)C_{M}\left(t\right)\cdot w.$$

Assume that w=e^jθ^, with$\theta =\arctan\left(\frac{A}{B}\right)$. The above formula can be rewritten as:

$$S_{L1}\left(t\right)=\sqrt{P_{c}}D_{c}\left(t\right)C_{c}\left(t\right)+j\sqrt{P_{p}}Multi\left(D_{p}\left(t\right)C_{p}\left(t\right),C_{L1CP}\left(t\right),D_{L1Cd}\left(t\right)C_{L1Cd}\left(t\right)\right)\;+\sqrt{P_{M}}D_{M}\left(t\right)C_{M}\left(t\right)\cdot e^{j\theta }.$$

It can be seen from the above equation that the C/A and P(Y) signals are modulated strictly according to the phase of 90°, and the M code and the other two signals can be separated on the baseband. With the signal modulated into the frequency band, we can see:

$$S_{L1}\left(t\right)=\sqrt{P_{c}}D_{c}\left(t\right)C_{c}\left(t\right)cos\left(2\pi f_{c}t+\theta_{0}\right)+\;j\sqrt{P_{p}}Multi\left(D_{p}\left(t\right)C_{p}\left(t\right),C_{L1CP}\left(t\right),D_{L1Cd}\left(t\right)C_{L1Cd}\left(t\right)\right)sin\left(2\pi f_{c}t+\theta_{0}\right)+\;\sqrt{P_{M}}D_{M}\left(t\right)C_{M}\left(t\right)\cdot e^{j\left(2\pi f_{c}t+\theta_{0}+\theta \right)}.$$

*f_c_* is the modulation carrier, and *θ*_0_ is the carrier initial phase. It can be clearly seen that the initial carrier phase of the L1M signal is *θ*_0_+*θ*, so it is speculated that the main reason why the constellation does not constitute a constant envelope is that the L1M signal and C/A signal is divided into two different transmission channel. For different links, the carrier frequencies used are the same, but the carrier phases are different.

The advantage of using this military civilian signal separation method is that the civilian signal ensures the multiplexing mode of the QPSK form, and avoids the degradation of the multiplexing efficiency or the cross-correlation between signals due to too many signal multiplexing paths, and guarantees the degree of distortion of the civilian frequency after passing through the power amplifier. The military M code signal is independently transmitted, which can ensure that the military signal is not affected by the intermodulation component generated by the mutual multiplexing of the civilian signal, and ensure that the military signal with adjusted power cannot be identified by the low-gain detection of civilian signals. So, it can meet the future demand for navigation warfare in the United States, that is, to improve the positioning accuracy of military applications without affecting the use of civilian users [16].

## 4. Conclusions

Through the actual acquisition and analysis of the new system L1 signal modulation and multiplexing method of the GPS III satellite, the following conclusions can be drawn:

1. The combined observation result of the new system signal is no longer the constant envelope modulation. The main reason is as follows. First, for users who only pay attention to the civilian frequency, the channel for transmitting the civilian signal still uses the UQPSK multiplexing method. Non-uniform weighted multiplexing is used to complete the in-phase branch multiplexing of L1C and P(Y) signals, which avoids the effective signal power degradation caused by the complex multiplexing mode and the influence between signals or signals and intermodulation components. Second, the satellite civil frequency and the M code signal are separately transmitted into two channels, and the initial phase of the generated carrier phase causes the superimposed civil frequency signal to generate a constellation shift phenomenon. Therefore, the combined signal observation result does not exhibit a constant envelope characteristic.
2. The power ratio of each component of the new system signal is adjusted. For the L1 frequency, the original L1C/A and L1P(Y) power ratio is reduced by half, and half of the remaining is reserved for the L1C signal, while the L1M signal ratio is unchanged. The advantage of this is that after adding new L1C signals that are compatible with other systems, the modern military signals will be further protected, and their anti-interference ability will be improved. Moreover, the M code signal is independently transmitted, which reduces the probability of detection and recognition of the enemy by means of the omnidirectional antenna civil navigation receiver when the power signal is adjusted for the military signal to meet the future demand for navigation warfare in the United States.

Considering that GPS III satellites have not yet officially provided services, the impact of different signal modulation and multiplexing methods on ranging and positioning performance has yet to be further analyzed and tested by GPS receivers. In addition, the possible adjustment of the new system signal modulation and multiplexing method requires further observation and research to provide reference for the next generation satellite navigation signal modulation design.

## Figures and Tables

**Table 1:** Ratio of L1 frequency signal power of two systems.

Signal Component	GPS III	GPS II
L1C/A	14.75%	28.37
L1P(Y)	8.17%	13.58%
L1M	25.32%	27.81%
L1Cp (BOC (1,1))	14.49%	0%
L1Cp (BOC (6,1))	3.22%	0%
L1Cd	5.80%	0%
total	71.75%	69.76%
